# Hématome post traumatique du muscle iléopsoas avec paralysie du nerf fémoral: à propos d'un cas et revue de la littérature

**DOI:** 10.11604/pamj.2015.20.198.6265

**Published:** 2015-03-03

**Authors:** Hicham Sallahi, Omar Margad, Adil Lamkhantar, Khalid Koulali idrissi

**Affiliations:** 1 Service de Traumatologie-Orthopédie, Hôpital Militaire Avicenne, Marrakech, Maroc; 2 Service de Traumatologie-Orthopédie, Hôpital Militaire Mohamed V, Rabat, Maroc

**Keywords:** Muscle psoas, paralysie, nerf fémoral, psoas muscle, paralysis, femoral nerve

## Abstract

L'hématome compressif du muscle *iliopsoas* dans le petit bassin est une complication connue des traitements anticoagulants, mais reste rare en post-traumatique. La présente observation illustre un cas de cet hématome chez un adolescent de 14 ans qui s'est présenté avec une douleur post-traumatique de la cuisse et un déficit actif d'extension de la jambe évoluant depuis plus de 3 mois. Un examen clinique a montré l'existence d'une paralysie complète du quadriceps. Une IRM du petit bassin a retrouvé un volumineux hématome du muscle *iliopsoas* comprimant le nerf fémoral. Un drainage chirurgical de l'hématome a été réalisé. La récupération musculaire était partielle après six mois de recul.

## Introduction

Le psoas est un muscle pair et symétrique, c'est le principal fléchisseur de la hanche [1]. Certaines lésions du psoas sont bien connues et ont déjà fait l'objet de descriptions. On peut citer notamment les abcès, les hématomes compliquant un traitement anticoagulant [2, 3] ainsi que les ostéochondroses du petit trochanter [4]. L'originalité de ce cas clinique résidait dans l’étiologie post-traumatique de l'hématome du muscle iliopsoas responsable de la compression du nerf, qui reste rare et peu décrite dans la littérature (16 cas en 40 ans) [5, 6].

## Patient et observation

**Figure 1:**
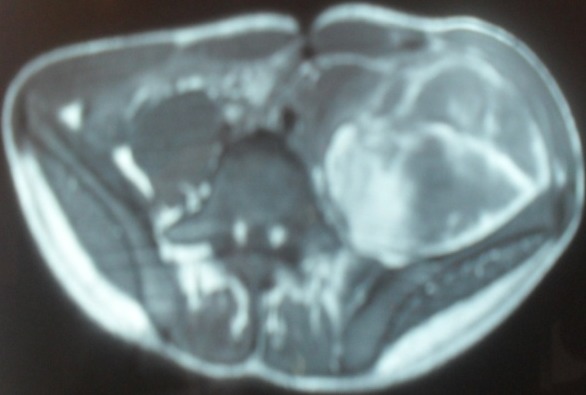
IRM de la hanche montrant un volumineux hématome du muscle psoas

**Figure 2:**
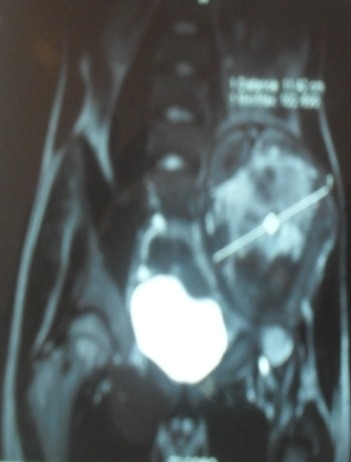
Coupes coronales de la même masse comprimant le nerf fémoral

Il s'agit d'un adolescent de 14 ans sans antécédent pathologique notable victime d'un accident de sport avec point d'impact au niveau de la hanche droite. L'examen clinique a montré l'existence d'une paralysie complète du muscle quadriceps avec un déficit sensitif de la face antérieure de la cuisse. Une radiographie de la hanche et du fémur homolatéral était normale. Une IRM de la hanche a révélé l'existence d'un volumineux hématome du muscle psoas iliaque comprimant le nerf fémoral (Figure 1, Figure 2). Le patient a bénéficié d'un drainage chirurgical de l'hématome avec repos, et d'une rééducation fonctionnelle pendant 45 jours. L’évolution a été marquée après six mois de recul par une indolence avec une mobilité normale de la hanche, une marche normale mais une récupération partielle du quadriceps.

## Discussion

L'anatomie du nerf fémoral permet d'expliquer cette paralysie par compression. Le fascia du muscle psoas-iliaque est épais et peu déformable [7]. Un hématome du muscle iliaque ne s’évacue pas spontanément et provoque une compression chronique du nerf fémoral le long de la gouttière du psoas-iliaque. En traumatologie sportive, plusieurs types sont rencontrés. Certaines lésions du corps charnu du psoas-iliaque survenant dans l'espace rétropéritonéal ou le bassin peuvent occasionner un important saignement et un hématome compressif. Il existe également des ruptures tendineuses basses qui peuvent être associées ou non à un arrachement osseux de l'enthèse sur le petit trochanter [8]. Le diagnostic est évoqué à l'interrogatoire et à l'examen clinique puis confirmé par la tomodensitométrie et/ou l'IRM. L'EMG, non systématique, permet habituellement d’évaluer la gravité de l'atteinte puis de suivre son évolution.

Dans la plupart des cas, les patients se plaignent d'abord de douleurs à l'aine ou au bassin. Les symptômes neurologiques apparaissent rapidement, en moyenne au cinquième jour post-traumatique. Dans de rares cas, les douleurs de l'aine sont isolées et aucun déficit neurologique n'est retrouvé [9, 10]. Chez ces patients, l'absence de signes compressifs du nerf fémoral s'explique probablement par la petite taille de l'hématome. D'après la littérature, le traitement est conservateur si le diagnostic est précoce et que la paralysie est partielle, mais il doit être chirurgical comme chez notre patient en cas de diagnostic tardif et surtout en cas de paralysie complète. Un seul cas retrouvé dans la littérature [9] présentant une paralysie totale a bénéficié d'un traitement conservateur mais avec persistance de séquelles sensitives à un an de recul. L'indication chirurgicale d’évacuation des hématomes post-traumatiques semble logique. Le retard dans l’évacuation chirurgicale de l'hématome pour décompresser le nerf fémoral peut conduire à une paralysie prolongée ou permanente [11, 12]. Dans la plupart des cas publiés, les patients ont été opérés entre la première et la quatrième semaine après le traumatisme avec une récupération totale entre six mois et un an [1]. Dans le cas rapporté, le diagnostic était tardif (3 mois) à cause des difficultés diagnostiques, et l’évacuation chirurgicale de l'hématome a permis une marche normale mais une récupération partielle de la fonction du quadriceps après six mois de recul.

## Conclusion

La pathologie traumatique du muscle iliopsoas est peu connue. Devant une radiographie normale, l’échographie ou l'IRM permettent souvent d'affirmer le diagnostic. Un geste chirurgical est souvent nécessaire pour prévenir une paralysie complète du nerf fémoral et permettre une récupération totale de la fonction du muscle quadriceps.
